# The integration of dilute acid hydrolysis of xylan and fast pyrolysis of glucan to obtain fermentable sugars

**DOI:** 10.1186/s13068-016-0612-0

**Published:** 2016-09-13

**Authors:** Liqun Jiang, Nannan Wu, Anqing Zheng, Zengli Zhao, Fang He, Haibin Li

**Affiliations:** 1 Guangdong Key Laboratory of New and Renewable Energy Research and Development, Chinese Academy of Sciences, Guangzhou Institute of Energy Conversion, Guangzhou, 510640 China; 2 Key Laboratory of Renewable Energy, Chinese Academy of Sciences, Guangzhou Institute of Energy Conversion, Guangzhou, 510640 China; 3 University of Chinese Academy of Sciences, Beijing, 100049 China

**Keywords:** Xylan, Glucan, Acid hydrolysis, Fast pyrolysis, Levoglucosan

## Abstract

**Background:**

Fermentable sugars are important intermediates in the biological conversion of biomass. Hemicellulose and amorphous cellulose are easily hydrolyzed to fermentable sugars in dilute acid, whereas crystalline cellulose is more difficult to be hydrolyzed. Cellulose fast pyrolysis is an alternative method to liberate valuable fermentable sugars from biomass. The amount of levoglucosan generated from lignocellulose by fast pyrolysis is usually lower than the theoretical yield based on the cellulose fraction. Pretreatment is a promising route to improve the yield of levoglucosan from lignocellulose.

**Results:**

The integration of dilute sulfuric acid hydrolysis and fast pyrolysis to obtain fermentable sugars was evaluated in this study. Dilute sulfuric acid hydrolysis could remove more than 95.1 and 93.4 % of xylan (the main component of hemicellulose) from sugarcane bagasse and corncob with high yield of xylose. On the other hand, dilute sulfuric acid hydrolysis was also an effective pretreatment to enhance levoglucosan yield from lignocellulose. Dilute acid hydrolysis could accumulate glucan (the component of cellulose) and remove most of the alkali and alkaline earth metals which were powerful catalysts during fast pyrolysis. Further increase in dilute acid concentration (from 0 to 2 %) in pretreatment could promote the yield of levoglucosan in fast pyrolysis. The acid pretreated sugarcane bagasse and corncob gave levoglucosan yields of 43.8 and 35.2 % which were obvious higher than those of raw sugarcane bagasse (12.0 %) and corncob (7.0 %).

**Conclusions:**

Obtaining fermentable sugars by combination dilute acid hydrolysis of xylan and fast pyrolysis of glucan could make full utilization of biomass, and get fermentable sugars economically from biomass for bio-refinery.

## Background

Sustainable production of fuels and chemicals is becoming increasingly important due to a growing global demand for energy, uncertainty in the supply of petroleum resources and environmental concerns with petrochemicals processing [1, 2]. Lignocellulose is one of the key potential and attractive energy resources to overcome increasing energy needs while being environmentally benign [3]. As the important intermediates in the biological and chemical conversion of biomass, fermentable sugars can be converted to a series of products or biofuels via microbial fermentation [4, 5]. Nevertheless, access to sugars is hindered by the recalcitrance of plant cell walls. The majority of glucose in lignocellulose is highly locked into crystalline cellulose. Conversion of biomass into sugars suitable for fermentation is one of the leading challenges in developing biofuels.

Enzymatic saccharification is generally considered to be a sustainable approach to release fermentable sugars from lignocellulose [6]. Enzyme is utilized to breakdown long-chain polysaccharides into oligosaccharides and monosaccharides. Several structural features, such as the surface area and crystallinity, have been proposed as major hurdles to both the rate and extent of biomass saccharification. Pretreatment prior to enzymatic hydrolysis is an essential step for overcoming the structural and steric barriers to enzyme access for more efficient hydrolysis. Enzymatic hydrolysis has several advantages, such as lower energy consumption, mild operating conditions, lower capital of equipment and no inhibitors or toxic derivatives contained in enzymatic hydrolysate. Nevertheless, high pretreatment and enzyme cost (accounting for one-third of the ethanol production cost from cellulose), slow hydrolysis rate, low product concentration and sensitivity to contaminants originated from other biomass components restrict its economical feasibility and large scale applications [7]. Since the final ethanol concentration is directly proportional to initial sugar concentration, to reduce the cost in cellulosic ethanol production, high sugar concentrations are required to achieve high ethanol titers to reduce the energy required for distilling the ethanol from fermentation broths [8]. However, the sugar concentration resulting from enzymatic saccharification is too low (about 1 %) for practical fermentation. As the solid loading increases, the viscosity of the hydrolysate also increases, contributing to mixing and mass transfer problems that reduce sugar conversion. High solid loading also leads to unproductive binding of enzyme to substrate and product inhibition, which are stumbling blocks for converting biomass to high concentrations of fermentable sugars. The technical barriers and fundamental limitations in enzymatic saccharification processes have proven to be complex and difficult to overcome [9]. Acid hydrolysis is also a common approach to hydrolyze biomass. The concentrated acids play a dual role in biomass hydrolysis. By disrupting its network of intra- and inter-chain hydrogen bonds, strong acids decrystallize cellulose and make it accessible to reagents, and by catalyzing the hydrolysis of glycosidic bonds, strong acids cleave hemicellulose and cellulose into sugars. Nonetheless, the hazards of handling concentrated acids and the complexities of recycling them have limited the adoption of this technology. In dilute acid, hemicellulose and amorphous cellulose are easily and nearly completely hydrolyzed to fermentable sugars accessible to microorganisms for biofuels production. But the remained solid residue (mainly contained crystalline cellulose) is more difficult to be hydrolyzed [10].

Although enzymatic saccharification and acid hydrolysis have received most of the attention, fast pyrolysis is a little-explored alternative technology to release fermentable sugars from biomass [11]. Cellulose readily depolymerizes during pyrolysis at 500 °C in very short residence time to yield predominately levoglucosan (1,6-anhydro-*β*-d-glucopyranose). The utilization of anhydrosugars is critical to ensure the economic viability of biofuel production from the pyrolysis of biomass because of the significant concentration of levoglucosan in the pyrolysis oil. Levoglucosan is the intramolecular glucoside between C-1 and C-6 of d-glucopyranose. Biochemical studies have indicated that levoglucosan could be directly phosphorylated to glucose 6-phosphate with the Mg-ATP-dependent levoglucosan kinase, and then metabolized through the general glycolytic pathway [12]. It has been proven that levoglucosan can be utilized as fermentation substrate. Itaconic acid was produced from pure levoglucosan by *Aspergillus terreus* K26 with the same yield and at the same rate as produced from glucose [13]. Citric acid was produced from levoglucosan by *Aspergillus niger* CBX-209 [14]. Oleaginous yeasts *Rhodosporidium toruloides* and *Rhodotorula glutinis* could be good candidates for pyrolytic sugar utilization with high conversion yield of levoglucosan to lipid production (similar to that of glucose) [15]. *Escherichia coli* KO11 was genetically modified and could utilize levoglucosan as a sole carbon and energy source for ethanol production [16]. As an anhydrosugar, levoglucosan could be readily hydrolyzed by mild acid to glucose, thereby providing another potentially rapid and efficient route to the production of bio-ethanol [17]. The feasibility of a bio-refinery concept in which levoglucosan present in bio-oil was separated, hydrolyzed, detoxified and fermented to produce ethanol or lipids was demonstrated [18]. Depending on the type of biomass and the operating conditions used for pyrolysis, pyrolysis oil could contain upwards of 33 wt% of levoglucosan [19]. Additionally, the process of fast pyrolysis which utilizes short time without enzymes or catalysts to overcome biomass recalcitrance and liberates valuable fermentable sugar, has a lower capital investment than that of biochemical pathways and shows a significant advantage from the economic perspective [20, 21].

Still, important challenge remains for implementation of biomass fast pyrolysis process to get fermentable sugar. Levoglucosan yields from cellulose can be as high as 59 % [22]. Lignocellulose fast pyrolysis produces a very low amount of levoglucosan when compared with the theoretical yield based on cellulose fraction [23]. Whole pyrolysis oil contains 3 % of levoglucosan when untreated wood biomass is pyrolyzed. The mineral content of biomass has significant catalytic effects on the pyrolysis process. Even trace level of certain ash components can alter both the thermal degradation rate and chemical pathways during pyrolysis. Alkali and alkaline earth metals (AAEM) strongly catalyze pyranose ring scission by forming coordinate bonds with the oxygen atoms of vicinal hydroxyl groups of the glucose ring. This leads to homolytic scission of the ring during pyrolysis resulting formation of light oxygenates, rather than levoglucosan. A negative correlation between total ash content and bio-oil yield has been demonstrated [24]. The catalytic effect of inorganic dopants on cellulose to produce levoglucosan was examined [25, 26]. Pure cellulose was doped with varying concentrations of AAEM (NaCl, KCl, MgCl_2_, CaCl_2_). Char formation was strongly enhanced by AAEM addition compared with untreated cellulose. Anion additives produced more levoglucosan than the AAEMs, but all were lower than the pure cellulose. The authors postulated that inorganic metals reduce the activation energy for reactions that form glycolaldehyde, formic acid and acetol directly from cellulose. Levoglucosan was, therefore, not a reactant to form these products, but rather the glucan reacted preferentially through alternate pathways as a result of inorganic addition, thereby reducing yields of levoglucosan. In terms of levoglucosan yield, the influence of the inorganic metal ions was in the order of K^+^ > Na^+^ > Ca^2+^ > Mg^2+^ [25, 26]. Ash content up to a certain critical degree significantly impacts the levoglucosan yield. It is essential to remove the ash below such critical concentration from biomass prior to fast pyrolysis to achieve a greater levoglucosan yield. A number of efforts have been made for demineralization to achieve a high yield of levoglucosan from lignocellulose such as hot water washing and glycerol pretreatment [23, 27, 28]. Levoglucosan yield can be greatly increased if acid pretreatment is applied to demineralize the feedstock prior to pyrolysis. Previous research showed that infusion of certain mineral acids (sulfuric acid, phosphoric acid, hydrochloric acid, nitric acid, acetic acid and formic acid) into biomass converted AAEM into thermally stable salts allowing cellulose to more readily thermally depolymerize to levoglucosan and the effect of sulfuric acid was better than others [22]. Although acid pretreatment has previously been identified as influencing the outcome of biomass pyrolysis, differences among dilute sulfuric acid concentration has not been investigated and the utilization of hemicellulose was ignored.

Herein, the effect of sulfuric acid was further investigated in this study. The objective of this work was to develop a method to obtain fermentable sugars by dilute acid hydrolysis integrated with a fast pyrolysis step. Sugarcane bagasse and corncob were chosen as the biomass, and several concentrations of dilute sulfuric acid were utilized. Sugarcane bagasse and corncob were first hydrolyzed by dilute sulfuric acid to remove the main component of hemicellulose (xylan) as sugars. The remained cellulose (glucan) in the solid residue was further fast pyrolyzed to get levoglucosan.

## Methods

### Raw material

Sugarcane bagasse was harvested from Dehong in Yunnan, China. Corncob was obtained from Baodi feed mill, Tianjin. Sugarcane bagasse and corncob were ground and sieved to the particle size range 0.11–0.18 mm and then dried in an oven at 105 °C until constant weights. Sulfuric acid was purchased from Chuandong Chemical Co. Ltd., Chongqing. The standard samples of glucose, xylose, levoglucosan, acetic acid, furfural and 5-hydroxymethylfurfural (5-HMF) were purchased from sigma (Shanghai).

### Elemental analysis

Carbon (C), hydrogen (H) and nitrogen (N) contents were measured with an organic elemental analyzer (Vario EL cube, Hanau, Germany). The contents of potassium (K), sodium (Na), calcium (Ca) and magnesium (Mg) were determined by an inductively coupled plasma optical emission spectrometry (ICP-OES) (Optima 8000, PerkinElmer, USA). For ICP-OES analysis, oven-dried (at 105 °C) biomass samples (0.3 g) were weighted into a test tube. The biomass was digested for 10 h in the 4 mL mixed acids of concentrated HNO_3_ and HClO_4_ (3:1, v/v). Then the digested sample was diluted to 10 mL with deionized water. In ICP-OES analysis, nebulizer flow was 1.5 L/min. The flush time, delay time and wash time were 10, 40 and 40 s, respectively. Five standard solutions of each metal were prepared and analyzed to generate external calibration curves for quantitative determination.

### Compositional analysis

The composition of carbohydrates was determined following the National Renewable Energy Laboratory (NREL) procedure [29]. Briefly, dried sample (0.3 g) was incubated with 3 mL of 72 % H_2_SO_4_ for 1 h at 30 °C with mixing. The mixture was diluted with 84 mL deionized water to a final acid concentration of 4 %. The solution was autoclaved for 1 h at 121 °C. The hydrolysate was filtered to separate the filtrate and solid residue. Calcium carbonate was used to neutralize the filtrate to pH 5–6. The sugars in the neutralized filtrate were analyzed by high performance liquid chromatography (HPLC, Waters 2695) with quantification referenced to standards, which were also autoclaved in 4 % H_2_SO_4_ to compensate for degradation. Glucose and xylose were separated by Aminex HPX-87P column (Bio-Rad, USA) at 80 °C with deionized water as mobile phase at a flow rate of 0.4 mL/min. Monosaccharides were detected by refractive index (RI) detector. The detector was operated at 50 °C. The samples were filtered through a 0.22 μm nylon filter before injection. The contents of glucan and xylan were determined from the concentration of the glucose and xylose, using an anhydro correction of a correction of 0.90 for glucose and 0.88 for xylose, respectively. Each sample was analyzed in triplicate.

### Thermogravimetric analysis (TGA)

TGA experiments were performed with a thermogravimetric analyzer (TGAQ50, TA, USA). The samples (4–6 mg) were loaded into an alumina crucible, and then were heated from 50 to 105 °C at a rate of 20 °C/min and held at 105 °C for 10 min. Consequently, samples were heated to 750 °C at a rate of 20 °C/min. Nitrogen was used as carrier gas (20 mL/min).

### Crystallinity measurement

Crystallinity of biomass before and after pretreatment was analyzed by X-ray diffraction (XRD) in X’Pert PROMPD X-ray diffract-meter (PANalytical V.B., Holland) employing Cu-Kα radiation. X-ray diffract-meter was set at 40 kV and 40 mA. Each sample (80 mg) was pressed into a lamellar container 20 mm in diameter and was scanned over diffraction angle (2θ°) of 5°–45° at a 0.01° per second of scanning rate by Cu radiation (λ = 1.54 Å). The percentage of crystalline material in the biomass was expressed as the crystallinity index (CrI), which was calculated by the equation following the procedure proposed by Segal [30]:

1 $$CrI = \frac{{ \, I {\text{002}}-I{\text{am}}}}{{ \, I 0 0 2 { }}} \, \times 1 0 0\;{\text{\% }}$$

where *I*_002_ was the intensity of the peak in crystalline phase (2θ = 22°) and *I*_am_ was the intensity of the peak in amorphous phase (2θ = 16°).

### Dilute acid hydrolysis

The dilute acid hydrolysis was performed in a 100 mL high pressure autoclave (HKY-3, Haian Petroleum Research Co. Ltd., Jiangsu, China). G0, G1, G2, G3, G4, G5 were used to denote as the un-pretreated (raw material), 0 % (hot water washing), 0.05, 0.5, 1 and 2 % dilute sulfuric acid pretreated sugarcane bagasse, respectively. C0, C1, C2, C3, C4, C5 were used to denote as the un-pretreated (raw material), 0 % (hot water washing), 0.05, 0.5, 1 and 2 % dilute sulfuric acid pretreated corncob, respectively. Sugarcane bagasse or corncob (3 g) was loaded in the 100 mL flasks containing 30 mL 0–2 wt % dilute sulfuric acid solution. The flasks were placed in a high pressure autoclave and reacted at 120 °C for 1 h. After hydrolysis, the solid phase and liquid phase were separated by filtration. Solid residue was washed with 300 mL distilled water to remove residual sulfuric acid, then freeze dried for 24 h (Boyikang Co., Ltd, Beijing). The pretreated biomass was then dried in an oven at 105 °C until constant weights. After drying, the pretreated feedstock was stored in sealed plastic containers for pyrolysis experiments. Concentration of glucose and xylose in dilute acid hydrolysate were determined by HPLC fitted with an Aminex HPX-87P column (Bio-Rad, USA) and RI detector. The concentration of acetic acid, furfural and 5-HMF in dilute acid hydrolysate were determined by HPLC fitted with an Aminex HPX-87H column (Bio-Rad, USA). The column and detector were operated at 60 and 50 °C, respectively. H_2_SO_4_ (5 mM) was utilized as mobile phase and the flow rate of the mobile phase was held constant at 0.6 mL/min. Acetic acid was analyzed by RI detector, while furfural and 5-HMF were analyzed by ultraviolet–visible (UV) detector at 280 nm. Compounds were identified and quantified by comparison to authentic standards using a five-point calibration curve. The hydrolysis yields of xylose and glucose were calculated as:

2 $${\text{Xylose yield (wt}} \%) = \frac{\text{ mass of xylose in the acid hydrolysate (g)}}{\text{ xylan mass of biomass (g) }} \, \times \;0.88\; \times 100 \; \%$$

3 $${\text{Glucose yield (wt}}\% ) = \frac{\text{ mass of glucose in the acid hydrolysate (g)}}{\text{ glucan mass of biomass (g) }} \, \times \;0.90\; \times 100\;\%$$

### Fast pyrolysis of biomass

Fast pyrolysis was conducted on a CDS pyroprobe 5200 series (CDS Analytical, USA), which was connected to a gas chromatograph/mass spectrometer (GC/MS) system (Agilent 7890 gas chromatograph, Agilent 7975C mass spectrometer, Agilent Technologies). The pyrolyzer used a heated filament to heat a quartz tube containing the sample. Sample (200–400 µg) weighted by a microbalance with an accuracy of 1 µg (XP6152, METTLER TOLEDO, Germany) was pyrolyzed during each test. The pyrolysis temperature, residence time and heating rate were fixed at 500 °C, 20 s and 10 K ms^−1^, respectively. The helium carrier gas continuously passed through the interface at a flow rate of 20 mL/min to transport the pyrolysate from the quartz tube into 240 °C GC injection port. The interface line between the pyrolyzer and GC maintained at 300 °C to prevent condensation of vapors. The split ratio was 50:1. A HP-INNO wax capillary column (Agilent 19091 N-133, 30 m length, 0.25 mm ID, 0.25 µm film thickness) was utilized for the chromatographic separation of pyrolysis products. The GC oven temperature program: initial temperature was 50 °C, held for 2 min, heated to 90 °C at a rate of 10 °C/min, 4 °C/min to 129 °C, and then 8 °C/min to 230 °C with a dwell time of 29 min. Helium flow rate remained at 1 mL/min. The mass spectrometer was operated at 150 °C in an electron impact mode (70 eV) and the mass scanned from *m/z* 12 to 500. Compound identification was achieved by matching with NIST mass spectral data library. Compounds were quantified by comparison to authentic standards using a five-point calibration curve. All experiments were tested in triplicate and averaged for quality assurance to compensate experimental reproducibility. The yields of main pyrolysis products were calculated based on the dry weight of solid sample in pyrolysis experiment. The potential yield of levoglucosan was experimentally observed maximum yield of levoglucosan from pure cellulose (59 wt%) [22]. The effectiveness was defined as the actual yield of levoglucosan from acid pretreated biomass divided by the potential yield of levoglucosan from the glucan contained in that biomass sample. The compound yield and effectiveness were calculated as:

4 $${\text{Compound yield (wt}}\%) = \frac{\text{ mass of compound (g)}}{\text{ mass of biomass (g) }} \, \times 1 0 0 \, \%$$

5 $${\text{Effectiveness}}\, (\% )= \frac{\text{actual levoglucosan yield}}{\text{ potential yield }} \, \times 1 0 0\, \%$$

## Results and discussions

### Component analysis in liquid hydrolysate and compositional analysis in solid residual

Various acid concentrations ranging from 0 to 2 % were impregnated for the pretreatment of sugarcane bagasse and corncob. The main components in the hydrolysate including glucose, xylose, acetic acid, furfural and 5-HMF were determined (Table 1). As the main component of hemicellulose in bagasse, xylan was hydrolyzed to xylose during the acid hydrolysis. The hydrolysis occurred together with the formation of by-products. The hydrolysis efficiency of xylan for sugar production was greatly enhanced by acid hydrolysis. The content of xylan and glucan in biomass before and after hydrolysis were also analyzed and listed in Table 2. After water washed, large amount of water-soluble fraction contained in biomass was removed and resulted in increase of biopolymers (glucan and xylan). An increase in the acid concentration (from 0 to 1.0 %), enhanced the formation of xylose (from 0.2 to 18.9 g/L). The content of xylan in the acid residue was decreased from 20.9 to 2.4 % gradually. The hydrolysis yield of xylose was increased from 1.0 to 89.9 %. When the acid concentration was 2 %, the concentration of xylose, the content of xylan and the hydrolysis yield of xylose were 18.0 g/L, 0.9 and 85.6 %, respectively. The decline of xylose concentration and xylose hydrolysis yield might attribute to the secondary decomposition of sugars at higher acid concentration. Moreover, with increasing acid concentrations, further decomposition of carbohydrates increased generation of inhibitors (furfural and 5-HMF). The concentrations of acetic acid (from 0.3 to 2.8 g/L), furfural (from <0.1 to 0.7 g/L) and 5-HMF (from <0.1 to 0.1 g/L) increased when acid concentration increased. Compared with xylose, the concentration of glucose (from 0.1 to 3.2 g/L) and the glucose yield (from 0.2 to 7.3 %) increased slightly. This phenomenon was mainly due to the easier hydrolysis of hemicellulose and amorphous cellulose than that of crystalline cellulose. Using 2 % dilute acid hydrolysis could remove more than 95.1 % of xylan and thus the pretreated sugarcane bagasse had the highest glucan content (65.6 %).

**Table 1:** Component analysis in dilute acid hydrolysate after pretreatment

Samples	Acetic acid (g/L)	Furfural (g/L)	5-HMF (g/L)	Xylose (g/L)	Glucose (g/L)
G1	0.3	<0.1	<0.1	0.2	0.1
G2	1.5	<0.1	0.1	8.4	1.3
G3	1.9	0.1	0.1	16.6	1.7
G4	2.4	0.2	0.1	18.9	2.7
G5	2.8	0.7	0.1	18.0	3.2
C1	0.5	<0.1	<0.1	0.2	0.1
C2	1.6	<0.1	<0.1	9.1	0.1
C3	2.6	0.1	<0.1	23.4	1.0
C4	2.9	0.2	<0.1	26.0	2.2
C5	3.0	0.9	<0.1	26.4	3.0

**Table 2:** Component analysis of biomass before and after pretreatment

Samples	Glucan (wt %)	Xylan (wt %)	Glucose yield (wt %)	Xylose yield (wt %)
G0	39.3	18.5	0	0
G1	50.6	20.9	0.2	1.0
G2	51.3	18.5	3.0	40.0
G3	60.9	5.7	3.9	79.0
G4	64.9	2.4	6.2	89.9
G5	65.6	0.9	7.3	85.6
C0	33.0	27.2	0	0
C1	37.8	33.3	0.3	0.6
C2	38.4	31.2	0.3	29.4
C3	56.6	6.2	2.7	75.7
C4	59.1	4.7	6.0	84.1
C5	61.1	1.8	8.2	85.4

Similar trends occurred for the samples of corncob treated with dilute acid hydrolysis. As acid concentration increased from 0 to 2.0 %, the concentration of xylose (from 0.2 to 26.4 g/L), acetic acid (from 0.5 to 3.0 g/L), furfural (from <0.1 to 0.9 g/L), and glucose (from 0.1 to 3.0 g/L) were increased. The concentration of xylose contained in corncob acid hydrolysate was higher than that of sugarcane bagasse hydrolysate. This phenomenon was mainly due to the higher content of xylan contained in corncob than that of sugarcane bagasse (27.2 vs. 18.5 %). Using 2 % dilute acid hydrolysis could remove more than 93.4 % of xylan and thus the pretreated corncob had the highest glucan content (61.1 %). Furfural and 5-HMF are inhibitors in fermentation, while their formation also means the loss of fermentable sugars. So, furfural and 5-HMF should be avoided. The by-products can be reduced using more moderate reaction conditions (such as, lower H_2_SO_4_ concentration, shorter reaction time and lower temperature).

### Elemental analysis of pretreated biomass

The elemental analysis of biomass pretreated by dilute sulfuric acid with different concentrations was shown in Table 3. No significant change of the composition of C, H and N in sugarcane bagasse (C 46.1–46.9 %; H 6.1–6.2 %; N 0.1–0.2 %) and corncob (C 44.5–45.1 %; H 6.0–6.1 %; N 0.2–0.3 %) were observed before and after pretreatment. The nitrogen content of sugarcane bagasse was lower than that of corncob. The AAEM consisting of K, Na, Ca and Mg were also experimentally determined. In this study, the dilute acid pretreatment resulted in steep decline in the content of AAEM. The total content of AAEM contained in sugarcane bagasse was reduced from 3403.5 to 673.9 ppm, and that of corncob was declined from 8994.6 to 1036.1 ppm. Using 0 % acid pretreatment (hot water washing) pretreatment could remove 98.8 and 98.2 % K from sugarcane bagasse and corncob. A higher acid concentration of pretreatment led to further removal of K from biomass. As for the composition of the volatile organics, K was found to promote depolymerization/fragmentation reactions to form lower molecular weight oxygenates at the expense of levoglucosan and other anhydrosugars [31]. Na, Ca and Mg had lower content in original materials and were more difficult to be removed compared with K. After 2 % acid pretreatment, 39.1 % of Na, 60.6 % Ca and 68.9 % Mg were removed from sugarcane bagasse, and 53.9 % of Na, 48.7 % Ca and 85.2 % Mg were removed from corncob.

**Table 3:** Elements of biomass before and after pretreatment

Samples	C (%)	H (%)	N (%)	K (ppm)	Na (ppm)	Ca (ppm)	Mg (ppm)	Total AAEM (ppm)
G0	46.6	6.1	0.2	1703.8	54.2	1462.0	183.5	3403.5
G1	46.1	6.1	0.2	20.3	53.6	1399.9	118.4	1592.2
G2	46.4	6.2	0.2	21.0	53.9	1181.2	112.5	1368.6
G3	46.5	6.2	0.1	12.2	58.8	866.9	86.1	1024.0
G4	46.9	6.1	0.1	10.6	32.2	763.3	68.9	875.0
G5	46.7	6.2	0.1	7.4	33.0	576.4	57.1	673.9
C0	44.9	6.0	0.3	6677.1	126.6	1554.0	636.9	8994.6
C1	44.5	6.0	0.3	120.8	70.8	1358.2	433.2	1983.0
C2	44.7	6.1	0.2	125.3	76.2	1320.4	440.6	1962.5
C3	44.6	6.0	0.2	124.8	69.0	779.6	207.8	1181.2
C4	45.1	6.1	0.2	109.1	73.1	807.4	120.8	1110.4
C5	45.1	6.0	0.2	86.5	58.4	797.1	94.1	1036.1

### Structural analysis of pretreated biomass

**Fig. 1:**
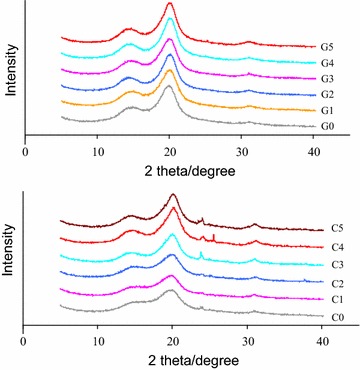
XRD analyses of lignocellulose before and after pretreatment

To further compare the crystallinity of the pretreated solids, XRD diffractograms of the raw and pretreated biomass were recorded as shown in Fig. 1. It was obvious that the intensities of (002) and (101) peaks were dramatically increased compared with those of raw materials. The calculated CrI for G0, G1, G2, G3, G4 and G5 were 51.1, 55.4, 56.4, 67.0, 71.4 and 72.1 %, respectively. The calculated CrI for C0, C1, C2, C3, C4 and C5 were 42.9, 49.3, 49.6, 63.1, 65.0 and 66.2 %, respectively. Dilute acid pretreated solids showed higher CrI than un-pretreated material, which was mainly due to the removal of amorphous cellulose and hemicellulose during pretreatment. The highest crystallinity was observed for 2 % dilute acid pretreated samples. The CrI seemed to be in direct proportion to glucan content. The role of cellulose crystallinity on pyrolysis reactions has not received enough attention. The crystallinity of cellulose decreased from 89.8 to 10.1 % as microcrystalline cellulose was pretreated by ball milling for 18 h. Accordingly, the yield of levoglucosan of pretreated cellulose decreased from 61.5 to 45.6 % [32]. The importance of a hydrogen bonding network in the reaction of cellulose pyrolysis was investigated [33]. It was found that the crystalline structure was maintained during levoglucosan formation. It had been postulated that amorphous cellulose led to more char and gas formation while crystalline cellulose contributed more to the formation of levoglucosan. Cellulose samples with a higher crystallinity tended to form levoglucosan in a higher yield [33]. However, the promoting mechanism of crystalline structure had not been fully validated.

### Thermal behavior of pretreated biomass

**Fig. 2:**
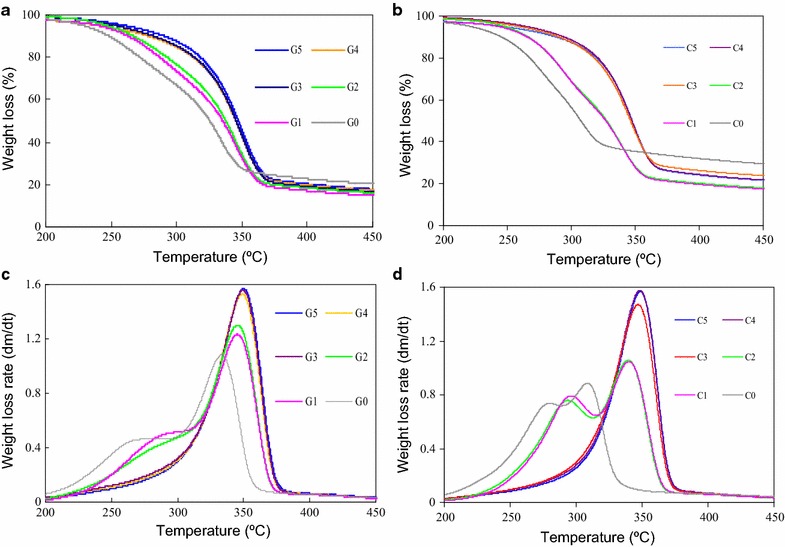
TG and DTG profiles of biomass. **a** TG profiles of pretreated sugarcane bagasse; **b** TG profiles of pretreated corncob; **c** DTG profiles of pretreated sugarcane bagasse; **d** DTG profiles of pretreated corncob

The weight loss curve was showed as thermogravimetry (TG) curve, and the weight loss rate was showed as differential thermogravimetry (DTG) curve, respectively (Fig. 2). The characteristic parameters of thermal degradation were presented in Table 4. The onset temperature of devolatilization (*T*_i_, corresponding to a weight loss of 5 % respect to the final weight loss) began at lower temperature for un-pretreated biomass, demonstrating that the solids were easily decomposed. The maximum DTG peak for cellulose decomposition changed little as shown by the value of *DTG*_max_. The value of *T*_max_ derived from TGA data represented the temperature at which the maximum decomposition rate occurred. Every biomass has a unique pyrolysis decomposition profile which was dependent on its lignocellulosic composition. Amongst the three components (hemicellulose, cellulose and lignin), hemicellulose is most reactive and decomposed at a lower temperature relative to lignin and cellulose. Cellulose has the lowest reactivity and highest decomposition temperature with a narrow temperature range of about 60 °C. Lignin decomposes over a wider temperature range overlapping the other two components. It demonstrated that the profiles of pretreated biomass had higher *T*_i_ and *T*_max_ compared with that of un-pretreated samples. Removal of minerals in biomass by pretreatment led to a shift of *T*_max_ to higher values and an enhanced thermal stability (Table 4). The dilute acid pretreatment could remove hemicellulose and accumulate crystalline cellulose, which might also impact the thermal degradation behavior. Thermal decomposition of lignocellulose presented two distinct peaks in the DTG due to the degradation of hemicelluloses, followed by cellulose. The initial shoulder at lower temperature was no longer present in sugarcane and corncob pretreated by dilute sulfuric acid, which also suggested that most of hemicellulose was removed in dilute acid hydrolysis.

**Table 4:** Characteristic parameters of biomass

Samples	Characteristic parameters			
	CrI (%)	*T*_i_ (°C)	*T*_max_ (°C)	DTG_max_ (%/min)
G0	51.1	228.5	333.3	1.1
G1	55.4	240.0	345.2	1.2
G2	56.4	252.7	349.5	1.6
G3	67.0	246.7	345.4	1.3
G4	71.4	240.9	349.0	1.5
G5	72.1	260.7	350.0	1.6
C0	42.9	225.0	307.9	0.9
C1	49.3	246.1	339.4	1.0
C2	49.6	249.6	339.1	1.0
C3	63.1	261.1	346.7	1.5
C4	65.0	265.6	348.0	1.6
C5	66.2	246.5	348.6	1.6

### Fast pyrolysis of pretreated biomass

Main compounds with relatively high content were identified and the yields of these compounds were calculated in Table 5. The fast pyrolysis pathway was hypothesized to proceed via a reaction intermediate known as “active cellulose” [34]. The active cellulose decomposed either through cleavage of glycosidic bonds that joined pyranose rings into cellulose chains or through fragmentation of the pyranose rings. The depolymerization of cellulose during pyrolysis resulted in the formation of levoglucosan. The first produced levoglucosan and the second generated oxygenates [25]. The subsequent decomposition of levoglucosan produced typical organic compounds such as 5-HMF, furfural, hydroxyacetone, hydroxyacetaldehyde, and some C_1_–C_2_ compounds. The production of levoglucosan was always lower in both raw materials. This result was a clear indication of undesirable effects that AAEM had on levoglucosan production. Raw sugarcane bagasse generated more levoglucosan than raw corncob due to its higher content of glucan and lower content of ash. The behavior of dilute acid pretreated samples was very different from the raw samples. In both cases, levoglucosan produced from the acid pretreated samples were significantly higher than that from the raw samples. Levoglucosan increased from 12.0 to 7.0 % of raw samples to 31.2 and 28.6 % after 0.05 % dilute acid pretreatment for sugarcane bagasse and corncob, respectively. Further increase in dilute sulfuric acid concentration increased the levoglucosan yield. The maximum in levoglucosan yield was obtained at 2 % sulfuric acid pretreated sugarcane bagasse (43.8 %) and corncob (35.2 %). Simultaneously, the yield of acetic acid and furfural were gradually diminished after dilute acid pretreatment, while the yield of 5-HMF increased. Acetic acid was a product from the thermal degradation of acetate groups in the hemicellulose. The decrease of acetic acid and furfural was likely due to the removal of hemicellulose. The increase of 5-HMF was likely attributed to the high content of glucan in dilute acid pretreated with sugarcane bagasse. Higher yields of acetic acid, furfural and 5-HMF were produced from corncob than sugarcane bagasse. On the other hand, the catalytic of AAEM in different samples had effect on the products distribution during biomass fast pyrolysis. As shown in Table 5, the effectiveness of pretreatment to enhance levoglucosan yields were greater than 11.9 % and ranged as high as 74.2 %. The experimental results showed that dilute acid hydrolysis could serve as an effective pretreatment method to improve the yield of levoglucosan. In the next work, the dilute acid hydrolysate and levoglucosan would be utilized as substrate for fermentation.

**Table 5:** Main compounds yield of biomass fast pyrolysis

Samples	Yield of compounds (wt %)					Effectiveness (%)
	Acetic acid	Furfural	5-HMF	Levoglucosan	Levoglucosan^a^	
G0	5.5	0.7	0.2	4.7	12.0	20.3
G1	4.5	0.7	0.4	13.0	25.7	43.6
G2	3.9	0.5	0.5	16.0	31.2	52.9
G3	3.1	0.5	0.6	23.8	39.1	66.3
G4	1.7	0.4	0.6	27.4	42.2	71.5
G5	1.4	0.4	0.6	28.7	43.8	74.2
C0	7.7	0.8	0.3	2.3	7.0	11.9
C1	6.3	0.7	0.4	8.3	22.0	37.3
C2	5.9	0.6	0.6	11.0	28.6	48.5
C3	2.9	0.6	0.6	19.3	34.1	57.8
C4	2.5	0.5	0.7	20.6	34.9	59.2
C5	2.2	0.5	0.8	21.5	35.2	59.7

^a^Data (%) were based on the mass of glucan

## Conclusions

The process to get fermentable sugars by integration of dilute acid hydrolysis and fast pyrolysis of sugarcane bagasse and corncob was evaluated. Dilute acid hydrolysis could remove more than 95.1 and 93.4 % of xylan obtaining 89.9 and 85.4 % yield of xylose from sugarcane bagasse and corncob, respectively. On the other hand, dilute acid hydrolysis prior to fast pyrolysis was also an effective pretreatment to enhance levoglucosan yield from acid pretreated biomass. Further increase in H_2_SO_4_ concentration (from 0 to 2 %) in dilute acid pretreatment increased the levoglucosan yield in fast pyrolysis. Compared with untreated feedstocks, the levoglucosan yields were increased from pretreated sugarcane bagasse (43.8 %) and corncob (35.2 %). The promotion was mainly attributed to the demineralization and accumulation of crystalline cellulose by acid pretreatment. The strategy in this work seemed a promising method to make full utilization of xylan and glucan, and get cost effective fermentable sugars from biomass for bio-refinery.

## Abbreviations

AAEM: alkali and alkaline earth metals
CrI: crystallinity index
DTG: differential thermogravimetry
GC/MS: gas chromatograph/mass spectrometer
5-HMF: 5-hydroxymethylfurfural
HPLC: high performance liquid chromatography
ICP-OES: inductively coupled plasma optical emission spectrometry
NREL: National Renewable Energy Laboratory
RI: refractive index
TG: thermogravimetry
TGA: thermogravimetric analysis
UV: Ultraviolet–visible
XRD: X-ray diffraction
